# Surveillance for *Ixodes scapularis* and *Ixodes pacificus* ticks and their associated pathogens in Canada, 2022

**DOI:** 10.14745/ccdr.v52i0102a04

**Published:** 2026-02-19

**Authors:** Gamal Wafy, Safa Ahmad, Christy Wilson, Heather Coatsworth, Jade Savage, Mark Nelder, Kirby Cronin, Pauline Zhang, Karine Thivierge, Kirsten Crandall, Priya Goundar, Louwrens Snyman, Emily Jenkins, Muhammed Morshed, Catherine Hogan, Min-Kuang Lee, Peter Buck, Annie-Claude Bourgeois, Salima Gasmi

**Affiliations:** 1 Centre for Food-borne, Environmental and Zoonotic Infectious Diseases, Public Health Agency of Canada, Ottawa, ON; 2 National Microbiology Laboratory Branch, Public Health Agency of Canada, Winnipeg, MB; 3 Department of Biology and Biochemistry, Bishop’s University, Sherbrooke, QC; 4 Public Health Ontario, Toronto, ON; 5 Laboratoire de santé publique du Québec, Sainte-Anne-de- Bellevue, QC; 6 Institute of Parasitology, McGill University, Sainte-Anne-de- Bellevue, QC; 7 Institut national de santé publique du Québec, Montréal, QC; 8 Ministry of Health, Regina, SK; 9 Department of Veterinary Microbiology, Western College of Veterinary Medicine, University of Saskatchewan, Saskatoon, SK; 10 Royal Alberta Museum, Edmonton, AB; 11 BCCDC Public Health Laboratory, BC Centre for Disease Control, Vancouver, BC; 12 Department of Pathology and Laboratory Medicine, University of British Columbia, Vancouver, BC; 13 Centre for Food-borne, Environmental and Zoonotic Infectious Diseases, Public Health Agency of Canada, Saint-Hyacinthe, QC

**Keywords:** *Ixodes scapularis*, *Ixodes pacificus*, surveillance, ticks, Borrelia, Anaplasma, Babesia, Powassan virus

## Abstract

**Background:**

This article continues the annual series on tick surveillance in Canada, tracking two of the primary tick vectors of concern in the country, *Ixodes scapularis* and *Ixodes pacificus*, which can transmit the agent of Lyme disease alongside several other tick-borne pathogens.

**Objective:**

This study analyzed passive and active tick surveillance data, including geographic distribution, pathogen prevalence and other characteristics to inform public health prevention.

**Methods:**

Passive and active surveillance data were compiled from eTick (an online, image-based platform), the National Microbiology Laboratory (Public Health Agency of Canada), provincial and local public health authorities and the Canadian Lyme Disease Research Network. Descriptive statistics of ticks and their associated pathogens are presented, including infection prevalence estimates.

**Results:**

In 2022, a total of 7,030 *I. scapularis* were submitted through passive surveillance from all provinces, while 911 *I. pacificus* were submitted from British Columbia (n=909) and Yukon (n=2). *Ixodes scapularis* submissions peaked in May and again in October. For *I. pacificus*, submissions peaked in May with a second, smaller peak in November. Six tick-borne pathogens (*Anaplasma phagocytophilum*, *Borrelia burgdorferi*, *Borrelia miyamotoi*, *Babesia microti*, *Babesia odocoilei*, Powassan virus) were identified from the *I. scapularis* collected by dragging in Manitoba, Ontario, Québec, New Brunswick or Nova Scotia.

**Conclusion:**

This report provides a summary of tick surveillance data collected in 2022. Tick characteristics and tick-borne pathogen infection prevalence were similar to previous years. Tick surveillance continues to play an important role in monitoring infection prevalence among ticks and their geographic distribution, which will help inform public health prevention and intervention efforts.

## Introduction

Tick-borne diseases (TBDs) continue to be a public health concern in Canada ((1)). *Ixodes scapularis* (blacklegged tick) and *Ixodes pacificus* (western blacklegged tick) are the primary tick vectors of importance in Canada, and are capable of transmitting several bacterial, viral and protozoan pathogens ((2,3)). These pathogens include *Borrelia burgdorferi* (senso stricto) (causing Lyme disease, LD), *Borrelia miyamotoi* (tick-borne relapsing fever), *Anaplasma phagocytophilum* (anaplasmosis), *Babesia* spp. (babesiosis) and Powassan virus–Lineage II ((2,3)). *Ixodes scapularis* are usually identified in Central and Eastern Canada and *I. pacificus* in British Columbia ((4–7)). In addition to LD becoming nationally notifiable in 2009, anaplasmosis, babesiosis and Powassan virus disease are nationally notifiable in humans as of 2024 ((8)).

Tick surveillance in Canada continues to play an important role in understanding the increasing risk of TBDs ((4)). By 2022, the national LD incidence had increased 6.5-fold relative to 2012 (from 338 to 2,525 cases) ((9)). Together, the increasing incidence of LD and the updated nationally notifiable status of other TBDs emphasize the importance of tick surveillance. Furthermore, *I. scapularis* ticks have been testing positive for *B. burgdorferi* since the 1990s ((4,5)). Continued surveillance efforts help identify the expanding geographic distribution of *I. scapularis* and their infection with relevant tick-borne pathogens ((10,11)). In addition, tick surveillance data can help inform prevention efforts such as where and when to target awareness campaigns.

Since 2019, *I. scapularis* and *I. pacificus* surveillance data collected in Canada has been summarized at the national level ((7)). These annual surveillance reports help monitor the current situation in Canada by summarizing the geographic distributions and seasonal activity of the selected *Ixodes* species ((6,7)). The objective of this surveillance report is to summarize characteristics of *I. scapularis* and *I. pacificus*, collected through passive and active surveillance in 2022. This article will also summarize the prevalence and spatial distribution of several tick-borne pathogens.

## Methods

### Data sources

This report uses passive and active tick surveillance data from seven organizations including public health authorities and academics. Passive surveillance data were provided by eTick (Bishop’s University), British Columbia Centre for Disease Control (BCCDC), Public Health Ontario (PHO), Saskatchewan Ministry of Health, Institut national de santé publique du Québec (INSPQ) and the National Microbiology Laboratory (NML) of the Public Health Agency of Canada. Active surveillance data were provided by the Canadian Lyme Disease Research Network (CLyDRN). The CLyDRN conducts active surveillance across all 10 provinces. In addition, active surveillance data was provided by BCCDC, PHO and INSPQ.

### Passive tick surveillance

This report follows similar methodology as previous annual reports ((6,7,12)). This analysis was limited to *I. scapularis* and *I. pacificus* collected in Canada in 2022. Submission records of ticks acquired outside of the submission province, as well as ticks acquired outside of Canada, were excluded, as were all records associated with a prior two-week history of travel outside of Canada. Ticks were either submitted individually (single submission) or in groups of two or more (multiple submission).

Since 2009, regional passive tick surveillance and testing programs have been gradually discontinued in several jurisdictions. This could be due, in part, to limited laboratory capacity as *I. scapularis* populations become established in various parts of the country and are more often encountered. eTick remains open to the public to submit images and spatiotemporal data of ticks encountered throughout Canada. eTick is a web-based, community-science project inviting the public to help with tick monitoring and used as a passive surveillance system for ticks in Canada ((13)). The images and associated data of ticks encountered by members of the public are submitted to the eTick website or through the eTick application, for identification by trained personnel. While images of *Dermacentor* ticks are only identified to genus, those of *Ixodes* and other genera are identified to species, unless the picture quality is inadequate in which case the specimen is requested to be submitted for further examination.

Ticks submitted from Saskatchewan and INSPQ were tested for *A. phagocytophilum*, *B. burgdorferi*, *B. miyamotoi* and *B. microti* using methods previously described ((7,14)). *Ixodes pacificus* submitted by the BCCDC were tested for *B. burgdorferi* ((15)). Ticks submitted from PHO were not tested for pathogens. Ticks submitted through eTick were not routinely requested for testing for tick-borne pathogens, but they could be forwarded onto a laboratory for this purpose at the request of local public health authorities.

### Active tick surveillance

Ticks were collected from the environment using drag sampling. This report analyzed *I. scapularis* collected via drag sampling from 19 sites in Alberta, 10 sites in Saskatchewan, 12 sites in Manitoba, 95 sites in Ontario, 124 sites in Québec, 10 sites in New Brunswick, 24 sites in Nova Scotia, five sites in Prince Edward Island and 10 sites in Newfoundland and Labrador. Also included in this report are *I. pacificus* data collected from 22 sites throughout British Columbia. Drag sampling took place in late spring/summer (May–July) across all regions. Some sentinel sites included a second sampling period in the fall (September–November).

Ticks submitted through CLyDRN and INSPQ were tested for *A. phagocytophilum*, *B. burgdorferi*, *B. miyamotoi*, *B. microti*, *B. odocoilei* and Powassan virus. *Ixodes pacificus* submitted by the BCCDC were tested for *B. burgdorferi*. Ticks that tested positive for *A. phagocytophilum* underwent further testing to identify the strain as pathogenic (*A. phagocytophilum–*human active, Ap-ha) or non-pathogenic (*A. phagocytophilum–*Ap-V1). The protocols for testing the ticks have been previously described ((7,14,16)).

### Analysis

**Descriptive and spatiotemporal characteristics:** For passive surveillance, descriptive statistics were calculated for several characteristics including submission type, the tick species covered in this report, province of acquisition, life stage (larva, nymph, adult female or adult male), level of engorgement (unfed or engorged), host (human, dog, cat or other) and month of collection. For active surveillance, descriptive statistics were calculated for province of collection and life stage (larva, nymph or adult). All data were cleaned and analysed in R (version 4.3.2) ((17)).

Ticks submitted through passive surveillance were mapped using QGIS (version 3.34.7) based on their location of acquisition. Records with a history of travel in Canada in the previous 14 days were geocoded to the probable location of exposure during travel. Data records were excluded from geocoding but kept in the overall analysis if the submitter had a history of multiple travel locations within Canada, travel to another province or were missing a location of tick acquisition. In active surveillance, the location of tick dragging was geocoded before being mapped except where site coordinates were already provided.

**Infection prevalence:** Prevalence was calculated as the number of positive ticks divided by the total number of ticks tested. Using the binom package in R, 95% confidence intervals were calculated.

## Results

### Overview of passive surveillance data

**Figure 1:**
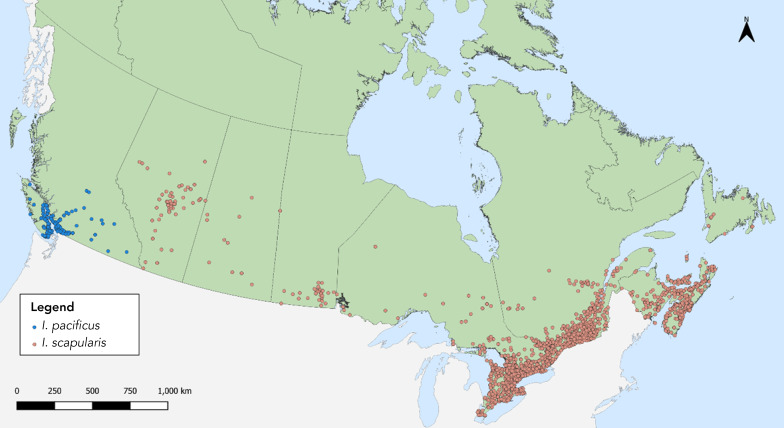
*Ixodes pacificus* and *Ixodes scapularis* submitted through passive tick surveillance, Canada, 2022^a^ ^a^ Each dot represents the probable location of acquisition for an *I. pacificus* (n=891) or *I. scapularis* (n=5,307) tick specimen submitted through passive surveillance. There were 20 *I. pacificus* and 1,723 *I. scapularis* that had locations that could not be geocoded or did not have any location information (missing data not shown in figure)

In 2022, there were 911 *I. pacificus* and 7,030 *I. scapularis* submitted from 10 provinces and one territory (**Table 1****, ****Figure 1**). No ticks were submitted from Northwest Territories or Nunavut. A higher proportion (54%) of the records were obtained from image-based submissions (n=4,324) while the rest were sample-based submissions (n=3,617). Submissions from Ontario, Québec and Nova Scotia comprised 83.4% of all ticks submitted. The majority (99.2%) of ticks were from single submissions, but there were 61 multiple submissions (range: 2–4 ticks per submission).

**Table 1:** Number of *Ixodes pacificus* and *Ixodes scapularis* submissions collected through passive surveillance by province, Canada, 2022

Province	Tick species (number of ticks)			Type of surveillance (number of ticks)^a^		Type of submission (number of submission)^b^	
	*Ixodes pacificus*	*Ixodes scapularis*	Total	Sample-based	Image-based	Single submissions	Multiple submissions
British Columbia	909	3	912	628	284	871	19
Alberta	0	126	126	0	126	126	0
Saskatchewan	0	17	17	0	17^c^	17	0
Manitoba	0	51	51	N/A	51	51	0
Ontario	0	4,338	4,338	2,211	2,127	4,249	42
Québec	0	1,629	1,629	778	851	1,629	0
Newfoundland and Labrador	0	12	12	0	12	12	0
New Brunswick	0	146	146	0	146	146	0
Nova Scotia	0	657	657	0	657	657	0
Prince Edward Island	0	51	51	0	51	51	0
Yukon	2	0	2	0	2	2	0
Total	911	7,030	7,941	3,617	4,324	7,811	61

^a^ Sample-based submissions are physical tick specimens; image-based submissions are images submitted to eTick

^b^ Single submissions consist of one tick; multiple submissions consist of two or more ticks submitted together by the same submitter

^c^ There were seven *I. scapularis* that were submitted through eTick and also tested for pathogens in Saskatchewan

Most ticks submitted were adult female (*I. pacificus*: 89.7%; *I. scapularis*: 89.9%) (**Table 2**). Adult males, nymphs and larvae were submitted less frequently (*I. pacificus*: 2.3%, 6.9% and 1.1%; *I. scapularis*: 5.6%, 4.4% and 0.1%, respectively). Overall, 11.3% of *I. pacificus* and 62.7% of *I. scapularis* adult females were engorged. Humans were the most common host among *I. pacificus* and *I. scapularis* (75.2% and 59.1%, respectively) followed by dogs (16.2% and 34.0%, respectively).

**Table 2:** Life stage, level of engorgement and host of *Ixodes pacificus* and *Ixodes scapularis* submitted through passive surveillance, Canada, 2022^a,b^

Characteristics	Tick species			
	*Ixodes pacificus*		*Ixodes scapularis*	
	n	%	n	%
**Life stage**				
Larva	10	1.1	4	0.1
Nymph	62	6.9	282	4.4
Adult female	810	89.7	5,701	89.9
Adult male	21	2.3	353	5.6
Total	903	100	6,340	100
**Level of engorgement^c^**				
**Adult female**				
Engorged	70	11.3	1,663	62.7
Unfed	548	88.7	991	37.3
Total	618	100	2,654	100
**Nymph**				
Engorged	N/A	N/A	74	63.8
Unfed	N/A	N/A	42	36.2
Total	N/A	N/A	116	100
**Host**				
Human	685	75.2	2,849	59.1
Dog	148	16.2	1,640	34.0
Cat	5	0.6	219	4.5
Other^d^	73	8.0	111	2.3
Total	911	100	4,819	100

Abbreviation: N/A, not applicable

^a^ Data are presented for all ticks where available, regardless of whether the tick was part of a single or a multiple submission

^b^ Life stage, level of engorgement and host were available for 99.1%, 68.6% and 100% of *I. pacificus*, respectively. Life stage, level of engorgement and host were available for 90.2%, 39.8% and 68.5% of *I. scapularis*, respectively. Ticks submitted only via eTick did not include information about level of engorgement (missing data not shown in table)

^c^ Level of engorgement was calculated for the adult female and nymph life stage. No engorgement data was available for *I. pacificus* nymphs

^d^ Includes environment, horse, goat, deer and bobcat

**Figure 2:**
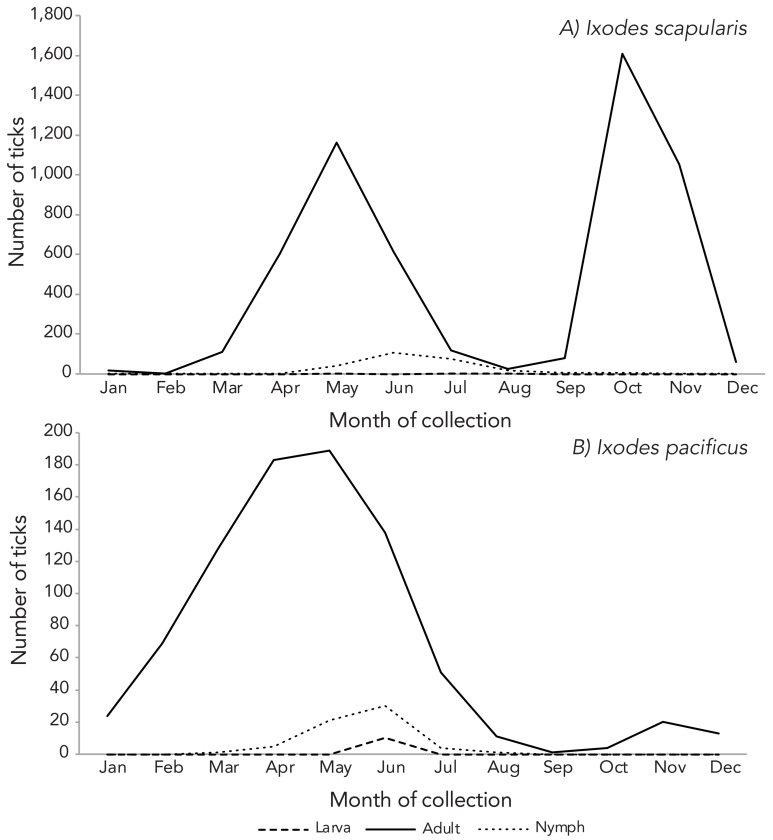
Number of *Ixodes scapularis* and *Ixodes pacificus* submitted through passive surveillance, by month and life stage, Canada, 2022^a,b^ ^a^ Available data are presented for A) *I. scapularis* (n=5,690) and B) *I. pacificus* (n=903) ticks submitted through passive surveillance ^b^ Month of acquisition and tick life stage data were available for 99.1% of *I. pacificus* and 80.9% of *I. scapularis* records (missing data not shown in figure)

Two submission peaks for adult *I. scapularis* were observed, one in May and a second slightly larger peak in October (**Figure 2**). The number of adult *I. pacificus* submissions peaked in May with a second, considerably smaller peak in November.

### Passive surveillance infection prevalence

The majority of *I. pacificus* submitted by BCCDC were tested for *B. burgdorferi* (n=624/625; 99.8%). In comparison, 64.6% (n=509/788) to 65.0% (n=512/788) of sample-based *I. scapularis* submissions were tested, depending on the pathogen of interest. Of 788 *I. scapularis*, three were submitted from BCCDC, seven from Saskatchewan and 778 from INSPQ. Although most of the prevalence data for *I. scapularis* came from Québec, one tick was found positive for *B. burgdorferi* in British Columbia, and another tick was found positive for *B. miyamotoi* in Saskatchewan (**Table 3**).

**Table 3:** Prevalence of *Borrelia burgdorferi* and *Borrelia miyamotoi* infection in physical specimens of *Ixodes pacificus* and *Ixodes scapularis* obtained through passive surveillance, by province and life stage, Canada, 2022

Province	Infection prevalence (% positive, 95% CI)					
	*Borrelia burgdorferi*			*Borrelia miyamotoi*		
	Adult	Nymph	Total	Adult	Nymph	Total
***Ixodes pacificus***						
British Columbia	7/624 (1.1, 0.5–2.3)	0	7/624 (1.1, 0.5–2.3)	NT	NT	NT
***Ixodes scapularis***						
British Columbia	1/3 (33.3, 0.8–90.6)	0	1/3 (33.3, 0.8–90.6)	NT	NT	NT
Saskatchewan	0/7 (0.0, 0.0–41.0)	0	0/7 (0.0, 0.0–41.0)	1/7 (14.3, 0.4–57.9)	0	1/7 (14.3, 0.4–57.9)
Québec	113/477 (23.7, 19.9–27.8)	2/25 (8.0, 1.0–26.0)	115/502 (22.9, 19.3–26.8)	5/477 (1.0, 0.3–2.4)	0/25 (0.0, 0.0–13.7)	5/502 (1.0, 0.3–2.3)
Total	114/487 (23.4, 19.7–27.4)	2/25 (8.0, 1.0–26.0)	116/512 (22.7, 19.1–26.5)	6/484 (1.2, 0.5–2.7)	0/25 (0.0, 0.0–13.7)	6/509 (1.2, 0.4–2.6)

Abbreviations: CI, confidence interval; NT, not tested

While ticks from human (95.7%) and non-human hosts (4.3%) were both tested, pathogens were found only in *I. scapularis* and *I. pacificus* submitted from human hosts. Pathogen testing information from Québec identified five ticks co-infected with *A. phagocytophilum* and *B. burgdorferi*, and one tick coinfected with *B. burgdorferi* and *B. miyamotoi* (**Table 4**).

**Table 4:** Prevalence of *Anaplasma phagocytophilum* and *Babesia microti* in physical specimens of *Ixodes pacificus* and *Ixodes scapularis* obtained through passive surveillance, by province and life stage, Canada, 2022

Province	Infection prevalence^a^ (% positive, 95% CI)					
	*Anaplasma phagocytophilum*			*Babesia microti*		
	Adult	Nymph	Total	Adult	Nymph	Total
***Ixodes pacificus***						
British Columbia	NT	NT	NT	NT	NT	NT
***Ixodes scapularis***						
British Columbia	NT	NT	NT	NT	NT	NT
Saskatchewan	0/7 (0.0, 0.0–41.0)	0	0/7 (0.0, 0.0–41.0)	0/7 (0.0, 0.0–41.0)	0	0/7 (0.0, 0.0–40.96)
Québec	13/477 (2.7, 1.5–4.6)	2/25 (8.0, 1.0–26.0)	15/502 (3.0, 1.7–4.9)	0/477 (0.0, 0.0–0.8)	0/25 (0.0, 0.0–13.7)	0/502 (0.0, 0.0–0.7)
Total	13/484 (2.7, 1.4–4.6)	2/25 (8.0, 1.0–26.0)	15/509 (3.0, 1.7–4.8)	0/484 (0.0, 0.0–0.8)	0/25 (0.0, 0.0–13.7)	0/509 (0.0, 0.0–0.7)

Abbreviations: CI, confidence interval; NT, not tested

^a^ Infection prevalence is presented as number positive/number tested (percentage positive, 95% confidence interval)

### Overview of active surveillance data

In 2022, *I. scapularis* (n=2,292) were collected in six provinces via active surveillance: Alberta (n=1), Manitoba (n=12), Ontario (n=904), Québec (n=1,077), New Brunswick (n=56) and Nova Scotia (n=242). Nymphs (n=1,116/2,292; 48.7%) were collected most often, followed by adults (n=894/2,292; 39.0%) and larvae (n=282/2,292; 12.3%). One *I. scapularis* was found in Alberta and it was not tested for pathogens. No *I. scapularis* were found by dragging in Saskatchewan, Prince Edward Island and Newfoundland and Labrador. *Ixodes pacificus* were collected in British Columbia (n=109). *Ixodes pacificus* nymphs (n=58/109; 53.2%) were collected most often, followed by larvae (n=26/109; 23.9%) and adults (n=25/109; 22.9%).

### Active surveillance infection prevalence

The most prevalent pathogen detected in *I. scapularis* was *B. burgdorferi* (24.4%, 95% CI: 22.5–26.4) (**Table 5**). *Anaplasma phagocytophilum* (2.9%, 95% CI: 2.2–3.7) was found in *I. scapularis* in all provinces where *I. scapularis* were recovered by active surveillance except Manitoba and Alberta (**Table 6**). Of the 57 ticks testing positive for *A. phagocytophilum*, 29 were tested to determine the strain variant, with 15 (51.7%) carrying the strain that is pathogenic to humans (Ap-ha). *Babesia odocoilei* was found in *I. scapularis* (10.6%, 95% CI: 9.3–12.1) in all provinces where active surveillance was conducted except Alberta (Table 6). The remaining pathogens (*B. miyamotoi*, *B. microti* and Powassan virus) were each found in less than 0.3% of tested ticks. Four *B. microti*-positive ticks were found in Québec and one in Nova Scotia (Table 6). Three *B. miyamotoi*-positive ticks were collected in Ontario and two in Québec (Table 5). Two ticks from Québec tested positive for Powassan virus Lineage II (Table 5). A higher infection prevalence was found in adult ticks versus nymphs for *B. burgdorferi*, *B. miyamotoi* and *A. phagocytophilum* (Table 5 and Table 6). In contrast, a higher infection prevalence was found in nymphs versus adult ticks for *B. microti* and *B. odocoilei* (Table 6).

**Table 5:** Prevalence of *Borrelia burgdorferi*, *Borrelia miyamotoi*, and Powassan virus in *Ixodes pacificus* and *Ixodes scapularis* obtained through active surveillance, by province and life stage, Canada, 2022

Province	Infection prevalence^a^ (% positive, 95% CI)								
	*Borrelia burgdorferi*			*Borrelia miyamotoi*			Powassan virus		
	Adult	Nymph	Total	Adult	Nymph	Total	Adult	Nymph	Total
***Ixodes pacificus***									
British Columbia	0/25 (0.0, 0.0–13.7)	1/58 (1.7, 0.0–9.2)	1/83 (1.2, 0.0–6.5)	0/9 (0.0, 0.0–33.6)	0/58 (0.0, 0.0–6.2)	0/67 (0.0, 0.0–5.4)	0/9 (0.0, 0.0–33.63)	0/58 (0.0, 0.0–6.2)	0/67 (0.0, 0.0–5.4)
***Ixodes scapularis***									
Manitoba	2/10 (20.0, 2.5–55.6)	0/2 (0.0, 0.0–84.2)	2/12 (16.7, 2.1–48.4)	0/10 (0.0, 0.0–30.9)	0/2 (0.0, 0.0–84.2)	0/12 (0.0, 0.0–26.5)	0/10 (0.0, 0.0–30.9)	0/2 (0.0, 0.0–84.2)	0/12 (0.0, 0.0–26.5)
Ontario	176/674 (26.1, 22.8–29.6)	70/223 (31.4, 25.4–37.9)	246/897 (27.4, 24.5–30.5)	3/676 (0.4, 0.1–1.3)	0/223 (0.0, 0.0–1.6)	3/899 (0.3, 0.1–1.0)	0/672 (0.0, 0.0–0.5)	0/223 (0.0, 0.0–1.6)	0/895 (0.0, 0.0–0.4)
Québec	43/113 (38.1, 29.1–47.7)	101/668 (15.1, 12.5–18.1)	144/781 (18.4, 15.8–21.3)	0/113 (0.0, 0.0–3.2)	2/668 (0.3, 0.0–1.1)	2/781 (0.3, 0.0–0.9)	1/113 (0.9, 0.0–4.8)	1/668 (0.1, 0.0–0.8)	2/781 (0.3, 0.0–0.9)
New Brunswick	7/16 (43.8, 19.8–70.1)	17/40 (42.5, 27.0–59.1)	24/56 (42.9, 29.7–56.8)	0/16 (0.0, 0.0–20.6)	0/40 (0.0, 0.0–8.8)	0/56 (0.0, 0.0–6.4)	0/16 (0.0, 0.0–20.6)	0/40 (0.0, 0.0–8.8)	0/56 (0.0, 0.0–6.4)
Nova Scotia	30/73 (41.1, 29.7–53.2)	38/163 (23.3, 17.1–30.6)	68/236 (28.8, 23.1–35.0)	0/73 (0.0, 0.0–4.9)	0/163 (0.0, 0.0–2.2)	0/236 (0.0, 0.0–1.6)	0/73 (0.0, 0.0–4.9)	0/163 (0.0, 0.0–2.2)	0/236 (0.0, 0.0–1.6)
Total	258/886 (29.1, 26.1–32.2)	226/1,096 (20.6, 18.3–23.1)	484/1,982 (24.4, 22.5–26.4)	3/888 (0.3, 0.1–1.0)	2/1,096 (0.2, 0.0–0.7)	5/1,984 (0.3, 0.1–0.6)	1/884 (0.1, 0.0–0.6)	1/1,096 (0.1, 0.0–0.5)	2/1,980 (0.1, 0.0–0.4)

Abbreviation: CI, confidence interval

^a^ Infection prevalence is presented as number positive/number tested (percentage positive, 95% confidence interval)

**Table 6:** Prevalence of *Anaplasma phagocytophilum*, *Babesia microti*, and *Babesia odocoilei* in *Ixodes pacificus* and *Ixodes scapularis* obtained through active surveillance, by province and life stage, Canada, 2022

Province	Infection prevalence^a^ (% positive, 95% CI)								
	*Anaplasma phagocytophilum*			*Babesia microti*			*Babesia odocoilei*		
	Adult	Nymph	Total	Adult	Nymph	Total	Adult	Nymph	Total
***Ixodes pacificus***									
British Columbia	1/9 (11.1, 0.3–48.2)	1/58 (1.7, 0.0–9.2)	2/67 (3.0, 0.4–10.4)	0/9 (0.0, 0.0–33.6)	0/58 (0.0, 0.0–6.2)	0/67 (0.0, 0.0–5.4)	0/9 (0.0, 0.0–33.6)	3/58 (5.2, 1.1–14.4)	3/67 (4.5, 0.9–12.5)
***Ixodes scapularis***									
Manitoba	0/10 (0.0, 0.0–30.8)	0/2 (0.0, 0.0–84.2)	0/12 (0.0, 0.0–26.5)	0/10 (0.0, 0.0–30.8)	0/2 (0.0, 0.0–84.2)	0/12 (0.0, 0.0–26.5)	2/10 (20.0, 2.5–55.6)	0/2 (0.0, 0.0–84.2)	2/12 (16.7, 2.1–48.4)
Ontario	27/676 (4.0, 2.6–5.8)	7/223 (3.1, 1.3–6.4)	34/899 (3.8, 2.6–5.2)	0/676 (0.0, 0.0–0.5)	0/223 (0.0, 0.0–1.6)	0/899 (0.0, 0.0–0.4)	32/676 (4.7, 3.3–6.6)	18/223 (8.1, 4.9–12.5)	50/899 (5.6, 4.2–7.3)
Québec	2/113 (1.8, 0.2–6.2)	13/668 (1.9, 1.0–3.3)	15/781 (1.9, 1.1–3.1)	0/113 (0.0, 0.0–3.2)	4/668 (0.6, 0.2–1.5)	4/781 (0.5, 0.1–1.3)	18/113 (15.9, 9.7–24.0)	104/668 (15.6, 12.9–18.5)	122/781 (15.6, 13.1–18.4)
New Brunswick	2/16 (12.5, 1.6–38.3)	3/40 (7.5, 1.6–10.4)	5/56 (8.9, 3.0–19.6)	0/16 (0.0, 0.0–20.6)	0/40 (0.0, 0.0–8.8)	0/56 (0.0, 0.0–6.4)	4/16 (25.0, 7.3–52.4)	9/40 (22.5, 10.8–38.5)	13/56 (23.2, 13.0–36.4)
Nova Scotia	0/73 (0.0, 0.0–4.9)	3/163 (1.8, 0.4–5.3)	3/236 (1.3, 0.3–3.7)	0/73 (0.0, 0.0–4.9)	1/163 (0.6, 0.0–3.4)	1/236 (0.4, 0.0–2.3)	11/73 (15.1, 7.8–25.4)	13/163 (8.0, 4.3–13.3)	24/236 (10.2, 6.6–14.8)
Total	31/888 (3.5, 2.4–4.9)	26/1,096 (2.4, 1.6–3.5)	57/1,984 (2.9, 2.2–3.7)	0/888 (0.0, 0.0–0.4)	5/1,096 (0.5, 0.1–1.1)	5/1,984 (0.3, 0.1–0.6)	67/888 (7.5, 5.9–9.5)	144/1,096 (13.1, 11.2–15.3)	211/1,984 (10.6, 9.3–12.1)

Abbreviation: CI, confidence interval

^a^ Infection prevalence is presented as number positive/number tested (percentage positive, 95% confidence interval)

**Figure 3:**
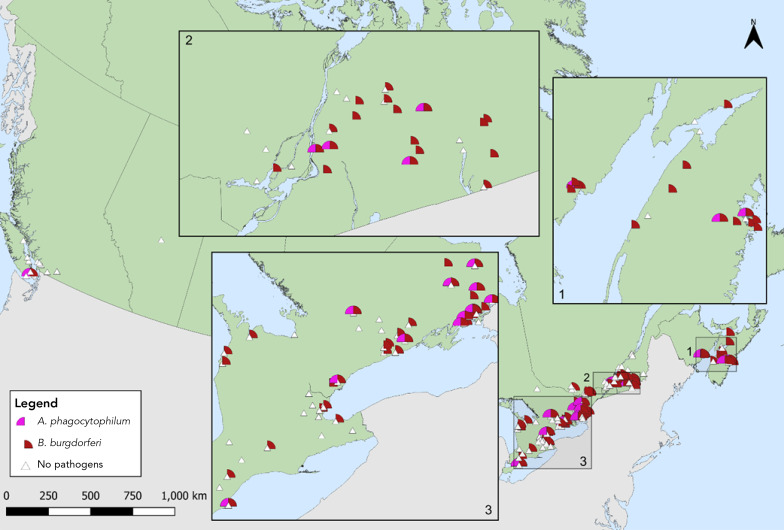
*Ixodes pacificus* or *Ixodes scapularis* with *Anaplasma phagocytophilum* or *Borrelia burgdorferi* collected through active surveillance, Canada, 2022^a^ Abbreviations: *A. phagocytophilum*; *Anaplasma phagocytophilum*; *B. burgdorferi*, *Borrelia burgdorferi* ^a^ Each symbol represents an active surveillance site where *A. phagocytophilum* (n=59) or *B. burgdorferi* (n=485) were found in *I. pacificus* or *I. scapularis*. There were 56 sites where no pathogens were detected. The inlays zoom in on regions in New Brunswick and Nova Scotia (Inlay 1), Québec and Ontario (Inlay 2) and Ontario (Inlay 3) where these ticks were found close together

**Figure 4:**
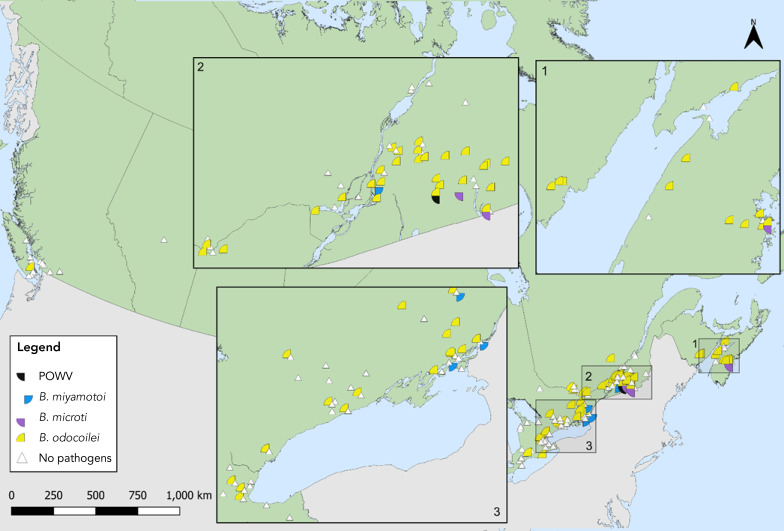
*Ixodes pacificus* or *Ixodes scapularis* with Powassan virus, *Borrelia miyamotoi*, *Babesia microti* or *Babesia odocoilei* collected through active surveillance, Canada, 2022^a^ Abbreviations: *B. microti*; *Babesia microti*; *B. miyamotoi*, *Borrelia miyamotoi*; *B. odocoilei*, *Babesia odocoilei*; *I. pacificus*, *Ixodes pacificus*; *I. scapularis*, *Ixodes scapularis*; POW, Powassan virus ^a^ Each symbol represents an active surveillance site where POW (Powassan virus) (n=2), *B. miyamotoi* (n=5), *B. microti* (n=5) or *B. odocoilei* (n=211) were found in *I. pacificus* or *I. scapularis*. There were 56 sites where no pathogens were detected. The inlays zoom in on regions in New Brunswick and Nova Scotia (Inlay 1), Québec and Ontario (Inlay 2) and Ontario (Inlay 3) where these ticks were found close together

Among *I. pacificus*, *B. burgdorferi* (1.2%, 95% CI: 0.0–6.5), *A. phagocytophilum* (3.0%, 95% CI: 0.4–10.4) and *B. odocoilei* (4.5%, 95% CI: 0.9–12.5) were found (Table 6). The site locations where *I. scapularis* and *I. pacificus* were collected in active surveillance and the pathogens detected therein are shown in **Figure 3** and **Figure 4**.

## Discussion

This annual summary provides an update on the characteristics, geographic distribution, and pathogen prevalence of *I. scapularis* and *I. pacificus* in Canada, previously analyzed in 2021 (unpublished data). In 2022, a total of 7,941 *I. pacificus* and *I. scapularis* were submitted through passive surveillance from 10 provinces and one territory.

Compared to 2021, infection prevalence estimates from passive surveillance for all pathogens tested are slightly higher across both tick species ((12)). Seven *I. pacificus* tested positive for *B. burgdorferi* from British Columbia (1.1% which is similar to the prevalence reported in 2021) (0.9%, 95% CI: 0.4–1.8). For *I. scapularis*, the prevalence of pathogens ranged from 0% to 22.7%, with *B. burgdorferi* being the most prevalent. A somewhat higher prevalence of *I. scapularis* infected with *B. burgdorferi* (22.7%, 95% CI: 19.1–26.5) was recorded in 2022 compared to 2021 (18.6%, 95% CI: 17.2–20.1).

Pathogen testing for passive surveillance specimens identified two distinct types of co-infections in Québec (*A. phagocytophilum* and *B. burgdorferi*; *B. burgdorferi* and *B. miyamotoi*), both of which have been reported in previous years ((6,7)). Adult ticks had a higher prevalence of infection across all pathogens except *A. phagocytophilum*; however, it is important to note that there was a considerably larger number of adult ticks collected and tested compared to nymphs in passive surveillance.

Passive surveillance data showed that tick life stage and month of collection continued to follow similar patterns as previous years ((6,7)); for example, a higher proportion of submitted ticks were adult females and found on human hosts. Furthermore, *I. scapularis* adult submissions showed a bimodal distribution in the month of collection with peaks occurring in May and October. This distribution has been consistently demonstrated to occur in Central and Eastern Canada through previous reports ((6,7,18–20)). *Ixodes pacificus* tick submissions were also consistent with previous reports, showing a large peak across April and May and a considerably smaller peak in November ((6,7,15)). These bimodal peaks reflect when ticks are most active due to suitable weather conditions. It is well recognized that Lyme disease symptom onset occurs around seasons where ticks are most active ((9,21,22)). In addition, tick engorgement, which reflects the ticks’ feeding activity, is slightly different compared to previous years, with adult female and nymphal *I. scapularis* showing a higher proportion of engorgement compared to previous years. This may also be due to the higher proportion of image-based submissions where engorgement data are missing.

Active surveillance data in 2022 identified six tick-borne pathogens (*A. phagocytophilum*, *B. burgdorferi*, *B. miyamotoi*, *B. microti*, *B. odocoilei*, Powassan virus) among the *I. scapularis* collected in Manitoba, Ontario, Québec, New Brunswick and Nova Scotia. The most prevalent pathogen was *B. burgdorferi* (24.4%, 95% CI: 22.5–26.4). The least prevalent pathogen was Powassan virus and was only identified in Québec. In contrast, three tick-borne pathogens (*A. phagocytophilum*, *B. burgdorferi* and *B. odocoilei*) were identified among *I. pacificus* collected in British Columbia.

Infection prevalence determined through active surveillance was comparable to previous years with slight differences depending on the pathogen ((6,7,12)). For example, the total prevalence for *B. burgdorferi* in *I. scapularis* increased from 22.3% in 2021 to 24.4% in 2022. To highlight, ticks in Ontario had a lower *B. burgdorferi* infection prevalence in 2022 (27.4%) compared to 2021 (29.3%), while ticks in Québec had a higher prevalence (18.4% in 2022 and 15.9% in 2021). *A. phagocytophilum* had a lower total infection prevalence in 2022 (2.9%) compared to 2021 (4.3%). *Babesia microti* had a higher total infection prevalence in 2022 compared to previous years. Finally, *B. odocoilei* had the highest infection prevalence in New Brunswick (23.2%) followed by Manitoba (16.7%) and Québec (15.6%). Infection prevalence should be interpreted with caution due to the varying number of ticks tested between provinces and years. Other factors that influence infection prevalence estimates from year-to-year or between provinces include variation in sites selected and their ecological and host-related characteristics ((23)).

## Strengths and limitations

In 2022, there were fewer pathogen testing data available for ticks collected through passive surveillance compared to previous years. This was in part due to the gradual discontinuation of passive surveillance programs over time; however, active surveillance data continued to provide standardized information on infection prevalence across Canada, with a larger number of sites and enhanced efforts to span more areas. Available passive surveillance data continued to provide comprehensive geographic and temporal information on *I. scapularis* and *I. pacificus*.

Other limitations included missing data across several tick characteristics such as life stage, which made interannual comparisons challenging. Furthermore, possible recall bias may have introduced uncertainty to existing passive surveillance data including tick location and date of collection. The annual variation in pathogen testing, including differing sample size and inconsistent surveillance site selection, reduced comparability of infection prevalence estimates. As additional annual data become available, interannual comparisons will be completed using appropriate statistical methods; therefore, comparisons to previous years were purely descriptive and did not assess statistical significance. Finally, this report provides an overview of tick surveillance across Canada but may not include all tick surveillance activities conducted in the country.

## Conclusion

Tick surveillance data in 2022 continued to highlight the characteristics of two important TBD vectors in Canada; *I. scapularis* and *I. pacificus*. *Borrelia burgdorferi* continued to be the most prevalent pathogen particularly in *I. scapularis*. The collection and spatiotemporal data, as well as infection prevalence data, were similar to previous years with slight increases or decreases depending on the pathogen and province.

These findings support public health initiatives such as public education on tick bite prevention and TBD risk area identification. Surveillance activity helps identify stable or shifting trends in pathogen prevalence or geographic distribution, especially as factors such as climate change and resulting expanding tick habitats are expected to continue to affect future tick and pathogen dynamics. Thus, continued investment in tick surveillance and prevention strategies will remain essential to reduce the burden of tick-borne disease on public health and the healthcare system.
